# Cooperative protein structural dynamics of homodimeric hemoglobin linked to water cluster at subunit interface revealed by time-resolved X-ray solution scattering

**DOI:** 10.1063/1.4947071

**Published:** 2016-04-14

**Authors:** Jong Goo Kim, Srinivasan Muniyappan, Key Young Oang, Tae Wu Kim, Cheolhee Yang, Kyung Hwan Kim, Jeongho Kim, Hyotcherl Ihee

**Affiliations:** 1 Department of Chemistry, KAIST, Daejeon 305-701, South Korea; 2 Center for Nanomaterials and Chemical Reactions, Institute for Basic Science (IBS), Daejeon 305-701, South Korea; 3 Department of Chemistry, Inha University, Incheon 402-751, South Korea

## Abstract

Homodimeric hemoglobin (HbI) consisting of two subunits is a good model system for investigating the allosteric structural transition as it exhibits cooperativity in ligand binding. In this work, as an effort to extend our previous study on wild-type and F97Y mutant HbI, we investigate structural dynamics of a mutant HbI in solution to examine the role of well-organized interfacial water cluster, which has been known to mediate intersubunit communication in HbI. In the T72V mutant of HbI, the interfacial water cluster in the T state is perturbed due to the lack of Thr72, resulting in two less interfacial water molecules than in wild-type HbI. By performing picosecond time-resolved X-ray solution scattering experiment and kinetic analysis on the T72V mutant, we identify three structurally distinct intermediates (I_1_, I_2_, and I_3_) and show that the kinetics of the T72V mutant are well described by the same kinetic model used for wild-type and F97Y HbI, which involves biphasic kinetics, geminate recombination, and bimolecular CO recombination. The optimized kinetic model shows that the R-T transition and bimolecular CO recombination are faster in the T72V mutant than in the wild type. From structural analysis using species-associated difference scattering curves for the intermediates, we find that the T-like deoxy I_3_ intermediate in solution has a different structure from deoxy HbI in crystal. In addition, we extract detailed structural parameters of the intermediates such as E-F distance, intersubunit rotation angle, and heme-heme distance. By comparing the structures of protein intermediates in wild-type HbI and the T72V mutant, we reveal how the perturbation in the interfacial water cluster affects the kinetics and structures of reaction intermediates of HbI.

## I. INTRODUCTION

Proteins are dynamic in nature, and their motions are directly related with their functions. To understand the function of a protein, it is essential to characterize the motions involved in structural transitions of the protein. While static structures of many proteins are well characterized with a sub-angstrom *resolution by* X-ray crystallography, it is difficult to elucidate their dynamic structures. A variety of time-resolved methods have been used to resolve the dynamic structures of proteins. Among them, time-resolved X-ray solution scattering (TRXSS), also known as time-resolved X-ray liquidography (TRXL), is relevant for probing structural dynamics of proteins in physiological solution phase and therefore can serve as a complementary technique to other structural probes used in structural biology. Over the last decade, TRXSS has been used to investigate photoinduced dynamics of various small molecules and proteins, elucidating their ultrafast structural dynamics.^1–20^

In various cellular processes, protein activities are often regulated by allostery,^21–27^ whereby the binding of an effector molecule at a site alters the reactivity of a distant active site. Homodimeric hemoglobin (HbI) is an excellent model system for investigating cooperative ligand binding and allosteric structural transition between two end states, the relaxed R state with a high ligand affinity and the tense T state with a low ligand affinity.^28–30^ Static and dynamic structures of HbI have been investigated by various experimental and theoretical methods such as time-resolved X-ray crystallography,^31–33^ time-resolved optical spectroscopies,^29,34–39^ nuclear magnetic resonance,^40^ and computational and molecular dynamics simulations.^41–43^ Among those techniques, time-resolved optical spectroscopies and time-resolved X-ray crystallography have been mainly used to study the dynamics of structural changes occurring in the allosteric structural transition of HbI. However, optical spectroscopies are generally not sensitive to global quaternary structural changes,^44–46^ and it has been reported that quaternary subunit rotation of HbI is attenuated in the crystalline phase.^31,32^ In this regard, TRXSS can provide complementary dynamic information on the allosteric structural transition of HbI in solution. Previously, we applied TRXSS to wild-type HbI and its F97Y mutant in solution.^14^ Kinetic and structural analyses of the TRXSS data revealed the kinetics and detailed structural changes among three intermediates involved in the structural transitions of HbI, for example, rate constants of R–T transition and CO recombination, quaternary rotation angles of subunits, change in the distance between two hemes, and the number of interfacial water molecules. Furthermore, we investigated the effect of mutation on the structural dynamics of HbI by performing TRXSS measurement on F97Y mutant HbI.

**FIG. 1.**
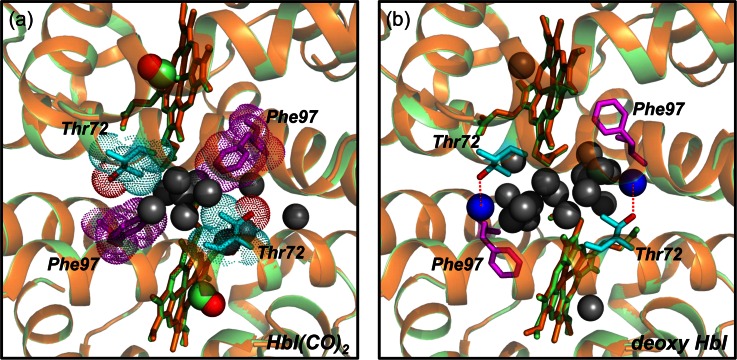
(a) Close-up view of the subunit interface of crystal structure of wild-type HbI(CO)_2_ (green color, pdb code: 3sdh) and T72V HbI(CO)_2_ (orange color, pdb code: 7hbi). Eleven interfacial water molecules are shown with grey spheres, and Thr72 and Phe97 are represented in sky blue and purple sticks, respectively. The hydroxyl group in the Thr72 is represented in red color. The methyl group of Thr72 in wild-type HbI packs against the extruded Phe97 of another subunit, stabilizing the liganded state via hydrophobic interaction between them. (b) Close-up view of the subunit interface of crystal structure of wild-type deoxy HbI (green color, pdb code: 4sdh) and deoxy T72V HbI (orange color, pdb code: 6hbi). In wild-type deoxy HbI, two interfacial water molecules represented by blue spheres are bound to hydroxyl group of Thr72 via hydrogen bonds. In contrast, in the T72V mutant, the lack of the hydroxyl group of Thr72 leads to two less water molecules than in the wild type.

In this work, we examine the role of interfacial water molecules in structural transition of HbI as an effort to extend our previous study on wild-type and F97Y mutant HbI. From crystallographic studies on wild-type HbI, the disruption of well-organized water cluster at the subunit interface was found to be one of the most notable structural changes upon ligand binding.^47,48^ The well-organized water cluster at the subunit interface of HbI is reorganized by the gain or loss of its constituent interfacial water molecules, depending on the ligated state of HbI, and plays a role of regulating the ligand binding affinity and cooperativity.^36^ For wild-type HbI, eleven water molecules constitute the water cluster in the CO-bound HbI and six additional water molecules participate in the cluster upon dissociation of the CO ligand.^36,47–50^ In particular, the hydroxyl group in the Thr72 residue forms a hydrogen bond with a water molecule (represented by blue spheres in Fig. 1(b)) in the cluster and stabilizes the unliganded state (T state) by enlarging the hydrogen-bonding network at the subunit interface. On the other hand, Thr72 is not hydrogen-bonded in the CO-liganded state (R state) and, instead, a methyl group in the residue packs against the extruded Phe97 of another subunit to stabilize the CO-liganded state.^51^ The water clusters in the R and T states of wild-type HbI are shown in Fig. 1. To investigate the role of the interfacial water cluster in the allosteric structural transitions and cooperative ligand binding of HbI in solution, we investigated the T72V mutant of HbI, which is isosteric to wild-type HbI but has a smaller water cluster than the wild type. Due to the lack of Thr72, the unliganded T state of the T72V mutant does not have any hydrogen bond with interfacial water molecules, leading to two less interfacial water molecules than in the wild type.^36,51^ The T72V mutant has served as a model system for studying the role of the interfacial water cluster. While the interfacial water network is altered as well in the F97Y mutant investigated in our previous work, the heme movement is also attenuated in that mutant, making it difficult to selectively examine the effect of interfacial water cluster on the structural transition of HbI. Thus, the T72V mutant is more relevant for investigating the role of water cluster on the structural dynamics of HbI. We performed the TRXSS experiment on the T72V mutant in solution and extracted the solution-phase structures of the intermediates and determined kinetic parameters based on a common kinetic model applicable to wild-type HbI, F97Y mutant, and T72V mutant. In particular, we obtained the distances between E and F helices, quaternary rotation angles of subunits, and heme–heme distances in the intermediates of T72V HbI, and quantitatively compared these structural parameters with those of wild-type HbI and F97Y HbI.

## II. TIME-RESOLVED X-RAY SOLUTION SCATTERING EXPERIMENT

### A. Sample preparation

The T72V mutation was carried out into the native recombinant HbI gene using the EZchange^®^ mutagenesis kit (Enzynomics™) with the following primers encoding T72V mutation: 5′-GGGCATTCCATCGTTCTGATGTACGCT-3′ and 5′-AGCGTACATCAGAACGATGGAATGCCC-3′(Genotech). The T72V mutant was over-expressed and purified as described for wild type.^52^ Carbonmonoxy derivatives of T72V dimeric hemoglobin mutant (T72V HbI(CO)_2_) solution were prepared as follows. A 2–4 mM deoxy T72V HbI solution in 100 mM phosphate buffer (pH 7) was prepared in a rubber-topped air-tight vial. The concentration of the protein solution was determined from the absorbance at 578 nm using the absorption coefficient of heme-oxygenated derivatives (14.3 mM^−1^ cm^−1^).^28^ The deoxy T72V HbI was reduced by adding 10 *μ*l of 1 M sodium dithionite solution to the deoxy T72V HbI solution under nitrogen atmosphere. The reduced samples were exposed to CO gas for 30 min to convert deoxy T72V HbI to the CO-bound T72V HbI(CO)_2_. The sample solutions were prepared just prior to X-ray solution scattering measurement. An aliquot of the resulting T72V HbI(CO)_2_ solution was transferred into a 1-mm diameter X-ray capillary (Hampton Research) and immediately sealed with epoxy to minimize gas exchange while CO gas was purged continuously into the capillary.

### B. Data acquisition

Time-resolved X-ray solution scattering data of the T72V mutant in solution were acquired at the 14IDB beamline at the Advanced Photon Source (APS). The experiment was performed following the pump-probe scheme. First, a sample solution of 3.0 mM concentration sealed in a capillary was excited by circularly polarized laser pulses of ∼35 ps duration and 532 nm center wavelength at a focal spot of 0.125 × 0.60 mm^2^ size. The power density of laser pulses was adjusted by a variable neutral-density (ND) filter, yielding the fluence of 0.5 mJ/mm^2^. A subsequent X-ray pulse was delivered with a time delay, Δ*t*, so that photo-induced structural changes of the protein were monitored through X-ray solution scattering patterns at time delays in the range from 100 ps to 10 ms. The X-ray pulses were generated at the APS with an energy spectrum peaked at 12 keV with a bandwidth of 4%. The X-ray pulses with the photon flux of ∼10^9^ photons were focused to a spot size of 0.09 × 0.07 mm^2^ at the sample position. Two-dimensional (2D) X-ray scattering patterns were collected using an area detector (MarCCD) with a sample-to-detector distance of 182 mm. The sealed capillary containing the sample was translated back and forth along its long axis with the movement synchronized with the pulse trains of laser and X-rays so that a fresh sample can be provided for every exposure of X-ray and laser pulses. Since 2D X-ray scattering images arising from the solution sample are generally centrosymmetric owing to random orientation of molecules in solution, one-dimensional (1D) scattering curves can be obtained by azimuthal integration as a function of the magnitude of the momentum transfer vector, q=(4π/λ) sin(2θ/2), without any loss of information. Resulting time-resolved 1D scattering curves measured at the time delay of Δ*t*, *S*(*q*, Δ*t*), contains scattering information arising from solute pairs, solvent pairs, and solute-solvent pairs. In solution, a majority of total scattering signal comes from solvent pairs or bulk solvent, and the signal from solute pairs of our interest is obscured by the solvent signal. To extract the underlying scattering signal from solute molecules, the scattering signal arising from bulk solvent is removed by subtracting a scattering curve measured at a negative time delay. As a result, we obtained time-resolved difference X-ray solution scattering curves, Δ*S*(*q*,Δ*t*), at the following time delays, which are spread evenly on a logarithmic time scale: −5 *μ*s, 100 ps, 178 ps, 316 ps, 562 ps, 1 ns, 1.78 ns, 3.16 ns, 5.62 ns, 10 ns, 17.8 ns, 31.6 ns, 56.2 ns, 100 ns, 178 ns, 316 ns, 562 ns, 1 *μ*s, 1.78 *μ*s, 3.16 *μ*s, 5.62 *μ*s, 10 *μ*s, 17.8 *μ*s, 31.6 *μ*s, 56.2 *μ*s, 100 *μ*s, 178 *μ*s, 316 *μ*s, 562 *μ*s, 1 ms, 1.78 ms, 3.16 ms, 5.62 ms, and 10 ms. The contribution from solvent heating was subtracted from the measured difference X-ray solution scattering curves as described in the supplementary material.^53^ During the data collection, the sample temperature was maintained at 25 °C with a cold nitrogen stream (Oxford Cryostream).

## III. KINETIC ANALYSIS

### A. Singular value decomposition (SVD) analysis

**FIG. 2.**
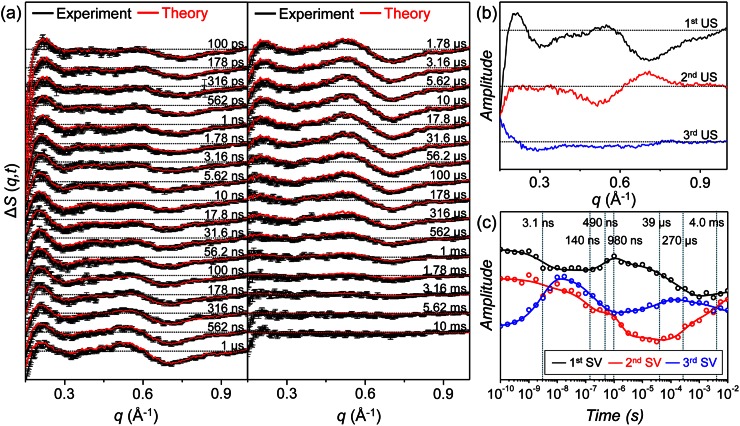
(a) Time-resolved X-ray solution scattering curves, Δ*S*(*q*,*t*), measured for T72V HbI in solution. Time-resolved X-ray solution scattering curves were measured at time delays from 100 ps to 10 ms (black curves) and were fit by theoretical scattering curves generated by a linear combination of left singular vectors (lSVs) obtained from kinetic analysis (red curves). (b) The first three significant lSVs obtained from singular value decomposition (SVD) analysis. (c) The first three right singular vectors (rSVs) fit by a sum of seven exponentials with time constants of 3.1 ns, 140 ns, 490 ns, 980 ns, 39 *μ*s, 270 *μ*s, and 4.0 ms.

Time-resolved difference X-ray solution scattering curves for the T72V mutant in solution were measured at time delays from 100 ps to 10 ms as shown in Fig. 2(a). As the protein undergoes structural transitions through various intermediates, the oscillatory patterns of the time-resolved scattering curves change. We performed kinetic analysis on the TRXSS data to determine intrinsic scattering patterns of the intermediates, so-called species-associated difference scattering curves, and time-dependent concentration changes of the intermediates governed by a certain kinetic model. To do so, we first applied singular value decomposition (SVD) analysis to the data matrix of which the columns are time-resolved difference scattering curves and the rows are the time delay points, and obtained left singular vectors (lSVs), right singular vectors (rSVs), and singular values. We can determine the number of structurally distinct intermediates from the number of singular vectors with significant singular values. The lSVs and rSVs provide a basis for the space spanned by the species-associated difference scattering curves of the intermediates and the time-dependent concentration changes of the intermediates, respectively. The relationship (i.e., transformation matrix) between the singular vectors and the real kinetic information (i.e., species-associated difference scattering curves and time-dependent concentration changes) can be found by a fitting process based on candidate kinetic models, which will be discussed in Sec. III B. However, rSVs themselves can provide the rates of transitions among the intermediates, because the transition kinetics are maintained upon the basis transformation. Therefore, global fitting of rSVs with a sum of exponential functions yields the rates of the transitions. We performed SVD analysis on the time-resolved scattering data of the T72V HbI in a *q*-range from 0.15 Å^−1^ to 1.0 Å^−1^ shown in Fig. 2(a) and found that only the first three significant lSVs and rSVs shown in Figs. 2(b) and 2(c), respectively, are meaningful, indicating that the initial T72V HbI(CO)_2_ in solution undergoes a photoreaction through three structurally distinct intermediates. We fitted the first three rSVs with a sum of seven exponentials and obtained time constants of 3.1 ns, 140 ns, 490 ns, 980 ns, 39 *μ*s, 270 *μ*s, and 4.0 ms. More details of the SVD analysis are described in the supplementary material.^53^

### B. Kinetic analysis

**FIG. 3.**
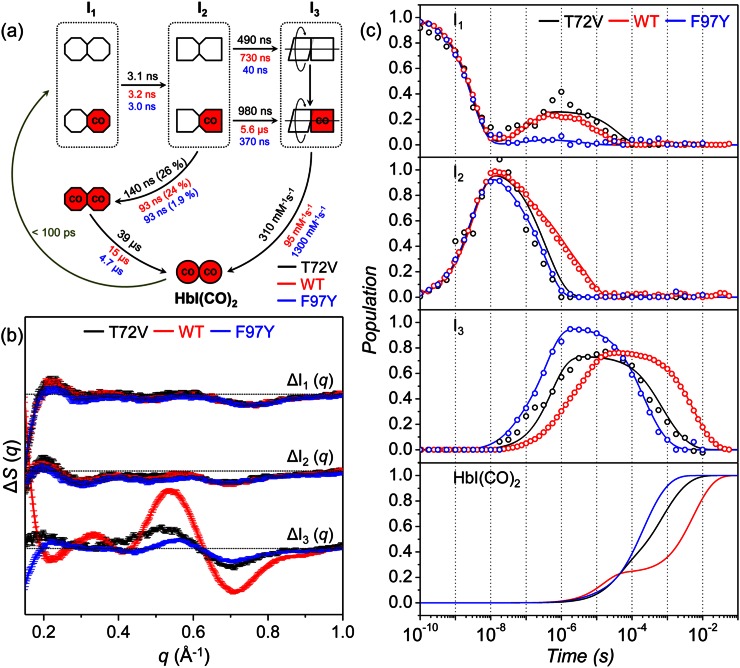
(a) Kinetic model for T72V HbI. Time constants for T72V, wild type, and F97Y are represented in black, red, and blue, respectively. The red (with “CO”) and white symbols represent ligated and photolyzed subunits, respectively. To indicate the change in tertiary structure with the progress of structural transition, we represented the subunits of each intermediate with symbols of different shapes. To indicate the change in quaternary structure in the transition from I_2_ to I_3_, we described the two subunits of I_3_ rotating with respect to each other. Two red octagons represent a ligated form of I_1_, which is formed by geminate recombination of CO with I_2_ and is structurally indistinguishable from the photolyzed forms of I_1_. (b) Species-associated difference scattering curves for the three intermediates of T72V (black), wild-type (red), and F97Y (blue) HbI. (c) Population changes of the three intermediates and HbI(CO)_2_ for T72V (black lines), wild-type (red lines), and F97Y (blue lines) HbI obtained by kinetic analyses. The open circles represent the optimized populations obtained by fitting the experimental difference scattering curves with the species-associated difference scattering curves of the three intermediates shown in (b). We note that the population of I_1_ rises back at ∼10 ns because geminate recombination of CO with the I_2_ intermediate leads to the formation of a ligated form of I_1_ (indicated by two red octagons in (a), which is structurally indistinguishable from the photolyzed forms of I_1_.

From the SVD analysis and subsequent fitting of rSVs, we identified three reaction intermediates and seven time constants for T72V HbI, which is in agreement with our previous TRXSS study on wild-type and F97Y HbI.^14^ Based on this information, we can construct a kinetic model that describes the mechanism of photoinduced structural transitions of T72V HbI. The most appropriate kinetic model for wild-type and F97Y HbI were determined by the following procedure. First, the latest two time constants (1.8 ms and 9.1 ms in the case of wild type) were combined to approximate a bimolecular time constant of CO recombination since the bimolecular kinetics cannot be fit by a single exponential for the protein solution (containing similar amounts of HbI and CO) used in our study. Considering the bimolecular CO recombination, the kinetic model should have five unimolecular transitions and one bimolecular transition among the three intermediates and the initial HbI(CO)_2_. The earliest time constant (3.2 ns for wild type) can be assigned to the transition from the first intermediate (I_1_) to the second intermediate (I_2_). The second time constant (93 ns for wild type) is quite similar to the dynamics of geminate recombination found in a study using transient absorption spectroscopy^34^ and thus was assigned to the geminate recombination of I_2_ leading to the formation of a ligated form of I_1_ (two red octagons in Fig. 3(a)). The next two time constants (730 ns and 5.6 *μ*s for wild type) were assigned to the biphasic transition from I_2_ to the last intermediate (I_3_), which will be interpreted to be the R-T transitions of two substates, fully photolyzed and partially photolyzed forms, as will be discussed later. The remaining time constant (15.2 *μ*s) was then assigned to the recovery of HbI(CO)_2_ from the ligated form of I_1_. The resultant kinetic model shown in Fig. 3(a) involves biphasic kinetics, geminate recombination, and bimolecular CO recombination among the three intermediates, which are termed as I_1_, I_2_, and I_3_ in the order of their appearance in time.

We applied this kinetic model to the TRXSS data of T72V HbI to test whether it can also explain the kinetics of T72V HbI. To do so, we generated theoretical difference scattering curves using time-dependent concentration changes of the intermediates expressed with a set of variable kinetic parameters of the kinetic model. Then, we optimized kinetic parameters by fitting the experimental difference scattering curves with the theoretical difference scattering curves. As a result, we obtained time-dependent concentration changes and species-associated difference scattering curves of the three intermediates. The suitability of the selected kinetic model can be evaluated by comparing the experimental scattering curves and the theoretical scattering curves generated from the kinetic analysis. The theoretical scattering curves are linear combinations of the first three lSVs, and the temporal changes of coefficients of the lSVs are governed by the kinetic model. Therefore, a satisfactory agreement between the experimental and theoretical scattering curves at all the time delays can be achieved only when the selected kinetic model is appropriate for describing the experimentally observed kinetics. As can be seen in Fig. 2(a), the theoretical time-resolved scattering curves generated from the kinetic analysis fit the experimental scattering curves well, assuring that the kinetic model shown in Fig. 3(a) is appropriate for the T72V mutant. Kinetic parameters determined from the kinetic analysis are presented in Fig. 3(a) and Table I. The species-associated difference scattering curves for the three intermediates are shown in Fig. 3(b), and the time-dependent concentration changes of the intermediates are shown in Fig. 3(c). We also tested other candidate kinetic models, but the best fit between the experimental and theoretical scattering curves was obtained with the kinetic model shown in Fig. 3(a), confirming that this kinetic model is the best one for T72V HbI.

**TABLE I.** Kinetic parameters obtained from kinetic analysis of TRXSS data of T72V HbI.

Parameter	Fit value	Error
Time constant for I_1_-to-I_2_ transition	3.1 ns	0.4 ns
Time constant for geminate recombination	140 ns	80 ns
Faster time constant for R-T transition (τ_RT1_)	490 ns	600 ns^a^
Slower time constant for R-T transition (τ_RT2_)	980 ns	1 *μ*s^a^
Time constant for formation of ligated I_1_	39 *μ*s	20 *μ*s
Rate constant for bimolecular CO recombination	310 mM^−1^s^−1^	7 mM^−1^s^−1^
Fraction of geminate recombination	26%	0.8%
Fraction of fully photolyzed forms	82%	3%

^a^ The error values of τ_RT1_ and τ_RT2_ for the T72V mutant are relatively large, because the values of τ_RT1_ and τ_RT2_ are close to each other. In fact, it is possible to approximate the kinetics of R-T transition for the T72V mutant even by a single exponential.

### C. Kinetics of T72V mutant HbI

In Figs. 3(a) and 3(c), we compared kinetic parameters and time-dependent concentration changes, respectively, of the intermediates for the T72V mutant HbI obtained from the kinetic analysis with those for the wild type and the F97Y mutant. According to the determined kinetic model, the initial T72V HbI(CO)_2_ is converted to the earliest intermediate, I_1_, within the experimental time resolution (∼100 ps), and I_1_ is transformed into the I_2_ intermediate with a time constant of 3.1 ± 0.4 ns, which is similar to the ones for the wild type (3.2 ns) and the F97Y mutant (3.0 ns), indicating that the I_1_-to-I_2_ transition is not much affected by either T72V or F97Y mutation. A part of the I_2_ intermediate undergoes geminate recombination with CO with a time constant of 140 ± 80 ns to form a ligated form of I_1_ (indicated by two red octagons in Fig. 3(a)), which is structurally indistinguishable from the photolyzed forms of I_1_. As a result, the population of I_1_ rises back after 10 ns as shown in Fig. 3(c). Subsequently, the ligated form of I_1_ returns to the initial T72V HbI(CO)_2_ with a time constant of 39 ± 20 *μ*s. In the T72V mutant, the geminate recombination is slower by 1.5 times than in the wild type and the F97Y mutant (93 ns for both) while the fraction of geminate recombination is similar as in wild-type HbI. The rest of the I_2_ intermediate is transformed to I_3_ biphasically with time constants of 490 ± 600 ns and 980 ± 1000 ns. The biphasic kinetics of the I_2_-to-I_3_ transition suggests that there exist two different substates of I_2_ and I_3_ although the two substates share a common species-associated difference scattering curve, that is, they are structurally indistinguishable from each other. In our previous TRXSS work on HbI,^14^ we showed that the branching ratio between the I_2_-to-I_3_ transitions starting from the two substates is dependent on the laser fluence, as was also observed in a study using transient absorption spectroscopy.^34^ Therefore, we can term those substates as fully photolyzed and partially photolyzed forms. In our previous TRXSS work on HbI,^14^ we showed that I_1_ and I_2_ have R-like structures and I_3_ has a T-like structure, and therefore, the transition from I_2_ to I_3_ corresponds to the R-T transition. The R-T transition is faster in the T72V mutant than in the wild type but slower than in the F97Y mutant as can be seen in the decay of I_2_ and the rise of I_3_ in Fig. 3(c). Then, the I_3_ intermediate returns to the initial T72V HbI(CO)_2_ via bimolecular CO recombination with a bimolecular rate constant of 310 ± 7 mM^−1^ s^−1^. The bimolecular rate constant increases in the order of wild type (95 mM^−1^ s^−1^), T72V (310 mM^−1^ s^−1^), and F97Y (1300 mM^−1^ s^−1^), which is in agreement with the order of the CO recombination rates determined by flash photolysis and equilibrium oxygen binding experiments.^29,34–36,38^

From the kinetics of structural transitions obtained from the kinetic analysis, we can examine the degree of cooperativity for the three types of HbI. Since the ligand dissociation triggers the R-T transition and thus can be regarded as the driving force of the R-T transition, the fully photolyzed I_2_ is likely to undergo faster R-T transition than the partially photolyzed I_2_ as depicted in Fig. 3(a). Therefore, the faster and slower time constants of I_2_-to-I_3_ transition can be assigned to the R-T transitions of fully photolyzed (τ_RT1_) and partially photolyzed (τ_RT2_) forms of I_2_, respectively. As the cooperativity between the subunits becomes higher, the separation in time scales for R-T transitions of fully and partially photolyzed forms will become smaller because the R-T transition of partially photolyzed form can be expedited by stronger interaction between the ligated and photolyzed subunits. Then, the ratio between the time constants (τ_RT2_/τ_RT1_) for the R-T transitions of partially photolyzed and fully photolyzed forms of I_2_ can serve as a measure of cooperativity. The τ_RT2_/τ_RT1_ ratio is 7.7 and 9.3 for the wild type and F97Y mutant, respectively, but it is only 2.0 for the T72V mutant. In other words, the τ_RT2_/τ_RT1_ ratio decreases in the order of F97Y > wild type > T72V. Since stronger interaction between the ligated and photolyzed subunits of HbI would result in a smaller value of τ_RT2_/τ_RT1_ ratio as discussed above, we can infer that the cooperativity increases in the order of F97Y < wild type < T72V. This prediction is in agreement with the varying trend of cooperativity in the three types of HbI obtained from the equilibrium oxygen binding experiments.^36,38^

## IV. STRUCTURAL ANALYSIS

### A. Species-associated difference scattering curves

From the SVD and kinetic analyses performed on the time-resolved X-ray scattering curves of the T72V mutant, we found that, for all the intermediates (I_1_, I_2_, and I_3_), both the fully photolyzed form and the partially photolyzed form are structurally indistinguishable from each other. This result provides direct evidence that the CO-bound subunit in the partially photolyzed form undergoes the same structural evolution as the photolyzed subunit, indicating the close interaction between the two subunits of HbI. Such symmetry between the tertiary structures of the subunits can account for the allostery of HbI, whereby the ligand binding state of one subunit affects the ligand binding affinity of another subunit. The tertiary structural symmetry of HbI in solution was also reported by studies using the molecular dynamics simulation and resonance Raman spectroscopy.^40,54^

We can get a glimpse of three-dimensional structures of the intermediates from the species-associated difference scattering curves for the intermediates. As shown in Fig. 3(b), the species-associated difference scattering curves of I_1_ and I_2_ for wild-type HbI, T72V HbI, and F97Y HbI are not much different from each other, suggesting that the structures of I_1_ and I_2_ are not affected by the T72V or F97Y mutation. However, the species-associated difference scattering curve of I_3_ changes significantly in the three types of HbI, indicating that the T72V and F97Y mutations alter the structure of I_3_. To distinguish the structures of I_3_ in the three types of HbI, we labeled I_3_ of the wild type, T72V HbI, and F97Y HbI as I_3_^WT^, I_3_^T72V^, and I_3_^F97Y^, respectively. We already obtained high-resolution structures of I_1_ and I_2_ for the wild type and F97Y HbI by performing the structure refinement aided by Monte Carlo simulations in our previous work.^14^ However, the structure of I_3_^T72V^ needs to be refined because its species-associated difference scattering curve is different from the structures of I_3_^WT^ and I_3_^F97Y^.

**FIG. 4.**
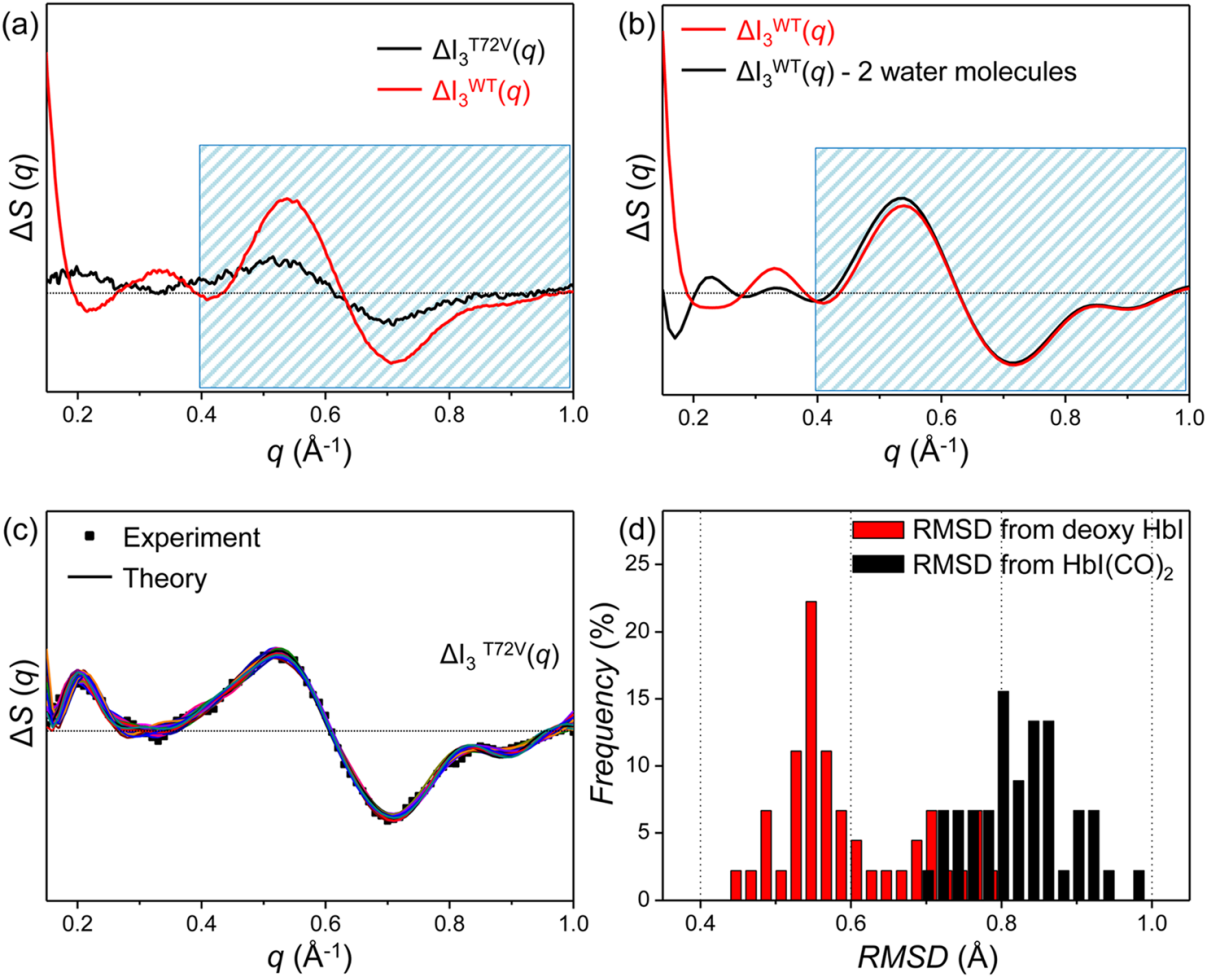
(a) Species-associated difference scattering curves of I_3_^T72V^ (black curve) and I_3_^WT^ (red curve). The T72V mutation alters scattering signal of I_3_ in solution significantly in a small-angle region as well as a wide-angle region represented by the blue shaded area. (b) Theoretical difference scattering curve calculated using a candidate structure of I_3_^WT^ (red curve)^14^ and difference scattering curve expected with two interfacial water molecules removed from I_3_^WT^ (black curve). The similarity of the scattering signals in a wide-angle range (blue shaded area) contrasts with the experimental observation shown in (a). (c) Species-associated difference scattering curve of I_3_^T72V^ (black dots) and theoretical difference scattering curves calculated from the candidate structures of I_3_^T72V^ (colored curves) obtained by the structure refinement. (d) The occurrence distribution of root-mean-square deviation (RMSD) calculated from the candidate structures for I_3_^T72V^ with respect to the crystallographic structures of deoxy HbI (red) and HbI(CO)_2_ (black).

Before performing the structure refinement for I_3_^T72V^, we can qualitatively explain the difference in species-associated difference curves of I_3_ for the wild type and the T72V mutant by considering the structural difference between crystallographic structures of the wild type and T72V HbI.^36,51^ Because I_3_ of the wild type has a T-like structure, we compared the crystallographic structures of T states (deoxy HbI) of the wild type (pdb code: 4sdh) and the T72V mutant (pdb code: 6hbi). According to crystallographic studies,^36,51^ the only noticeable structural difference between the T states of the wild type and the T72V mutant is the presence or absence of two interfacial water molecules that are hydrogen-bonded to Thr72 residues as shown in Fig. 1(b). To check whether the structural difference between wild-type HbI and its T72V mutant observed in the crystalline phase is maintained in solution, we theoretically calculated the difference scattering curve expected upon the loss of the two interfacial water molecules from I_3_^WT^. In Fig. 4(b), we compared the theoretical difference scattering curves calculated from the refined structure of I_3_^WT^ (red curve) in our previous study and the structure with two interfacial water molecules removed from the refined I_3_^WT^ (black curve). Specifically, we removed the two water molecules that were found to be lost in the deoxy form of the T72V crystal as shown with blue spheres in Fig. 1(b). The theoretical scattering curves were calculated using CRYSOL version 2.6.^55^ The scattering contribution of the interfacial water molecules was calculated explicitly by treating them as part of the heme group. As can be seen in Fig. 4(b), the loss of two water molecules affects the difference scattering curve only in a small-angle region (0.15 Å^−1 ^< *q* < 0.4 Å^−1^) and the features in a wide-angle region (0.4 Å^−1 ^< *q* < 1.0 Å^−1^) are nearly intact (blue shaded area in Fig. 4(b)). However, the species-associated difference scattering curves of I_3_^WT^ and I_3_^T72V^ determined from the TRXSS experiment are quite different from each other, even in the wide-angle region (blue shaded area in Fig. 4(a)). Therefore, it is expected that there are other structural differences between I_3_^WT^ and I_3_^T72V^ in solution besides the presence or absence of the two interfacial water molecules observed in the crystallographic studies. To investigate the details of structural difference between I_3_^WT^ and I_3_^T72V^ in solution, structure refinement is required.

### B. Structure refinement

We performed structure refinement using a species-associated difference curve of I_3_^T72V^ as was done for the structures of the intermediates in the wild type and the F97Y mutant.^14^ For the structure refinement, we applied a rigid-body modeling approach where the crystallographic structure of deoxy T72V HbI (pdb code: 6bhi) was used as the template structure.^5^ The rigid-body modeling approach can be used to determine the atomic-level structure of an intermediate when a crystallographic structure of the ground-state protein is available. The whole protein structure was described by eighteen rigid bodies with helices and hemes considered as basic units of the rigid bodies. The positions and orientations of the rigid bodies were refined based on a Monte Carlo simulation algorithm to minimize the discrepancy between the theoretical difference scattering curve calculated from the refined structure and the species-associated difference scattering curve of I_3_^T72V^ determined from the experiment. The refinement process was repeated for 600 different initial structures whose rigid bodies were randomly moved from the template structure. From the refinement of the initial structures, we selected 45 candidate structures that exhibit χ^2^ values (a quantified value of the discrepancy between the experimental and theoretical difference scattering curves) below a certain threshold. The theoretical difference scattering curves for the refined candidate structures are shown in Fig. 4(c). The overall structure of each I_3_^T72V^ candidate structure was evaluated by calculating root-mean-square deviation (RMSD) in the positions of C_α_ atoms with respect to the crystallographic structures of T72V HbI(CO)_2_ (pdb code: 7hbi) and deoxy T72V HbI (pdb code: 6hbi), yielding a RMSD distribution shown in Fig. 4(d). The average RMSD's for I_3_^T72V^ were found to be 0.60 ± 0.09 Å and 0.82 ± 0.06 Å with respect to deoxy T72V HbI and T72V HbI(CO)_2_, respectively, indicating that I_3_^T72V^ has a structure closer to deoxy T72V HbI than T72V HbI(CO)_2_. However, I_3_^T72V^ in solution and deoxy T72V HbI in crystal are still structurally different from each other with RMSD as large as 0.60 Å.

### C. Structural parameters of the intermediates

**FIG. 5.**
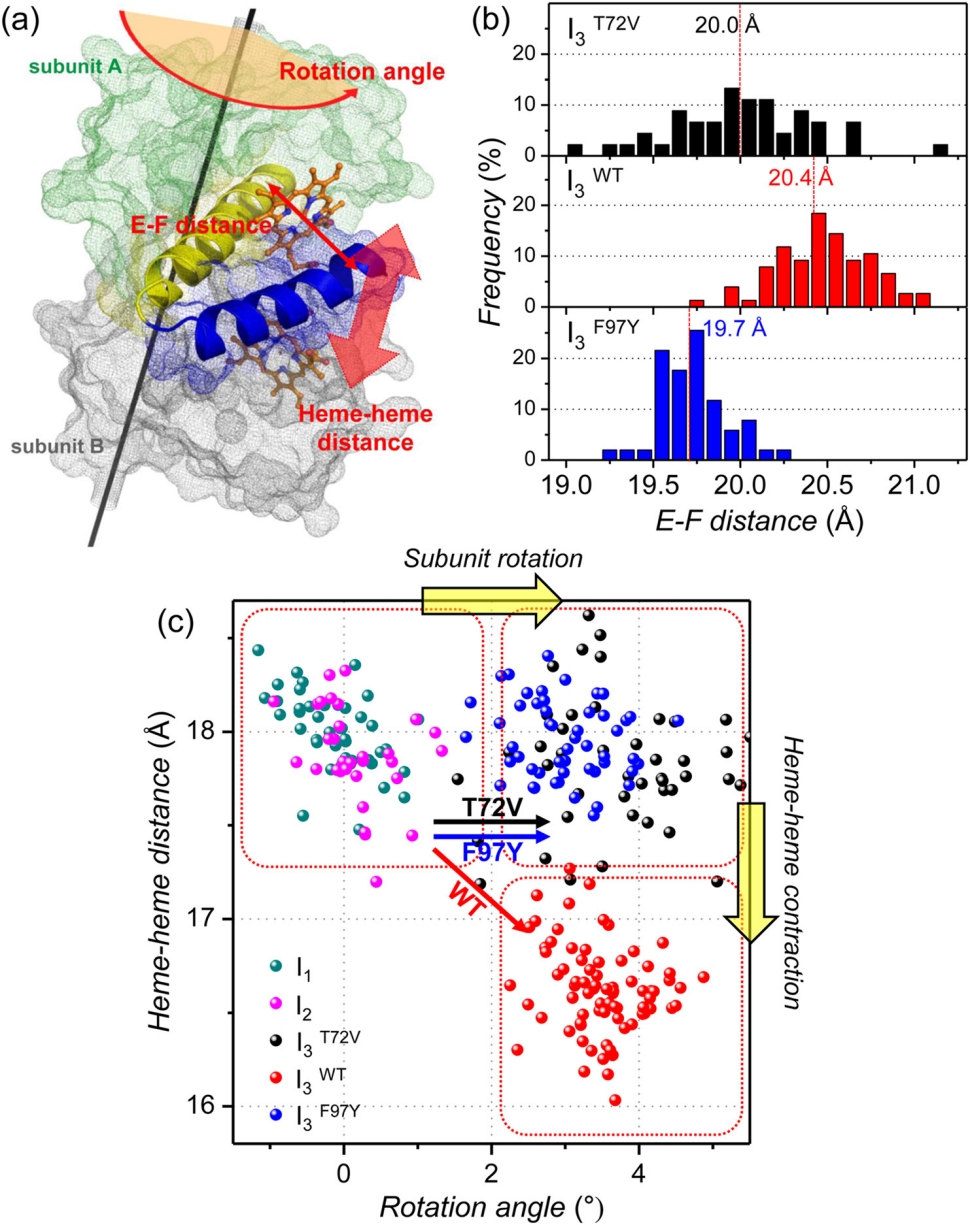
(a) Illustration of structural parameters inspected in candidate structures of various intermediates. E and F helices are represented in yellow and blue, respectively, and two hemes are shown in orange sticks. The rotation axis is shown with the black line. (b) The occurrence distribution of E-F distances calculated for I_3_^T72V^ (black), I_3_^WT^ (red), and I_3_^F97Y^ (blue). (c) Heme-heme distances in the candidate structures of the intermediates plotted as a function of subunit rotation angle. The dots in dark cyan, magenta, black, red, and blue correspond to candidate structures of I_1_, I_2_, I_3_^T72V^, I_3_^WT^, and I_3_^F97Y^, respectively. The black, red, and blue arrows represent the I_2_ to I_3_ transition of T72V, wild-type, and F97Y HbI, respectively. Upon photoinduced structural transition, wild-type HbI undergoes both subunit rotation and heme-heme distance contraction whereas only subunit rotation occurs in T72V and F97Y mutants.

To extract detailed structural information from the refined candidate structures for the intermediates, we examined several structural parameters illustrated in Fig. 5(a). First, we calculated E-F distance, which is defined by the distance between C_α_ atoms in Leu66 and Ile102 residues, for the candidate structures of I_3_^WT^, I_3_^T72V^, and I_3_^F97Y^. In a recent study, meta-analysis for a large collection of static and time-resolved structures of wild-type HbI and its mutants suggests that the E-F distance is related to the ligand binding affinity. For example, T states with low binding affinity exhibit larger E-F distance than R states with high binding affinity.^33^ Based on this result, the E-F distance can be used as a measure of ligand binding affinity of an intermediate. The occurrence distributions of the E-F distance for I_3_^WT^, I_3_^T72V^, and I_3_^F97Y^ in Fig. 5(b) show that the average E-F distance becomes shorter in the order of I_3_^WT^ (20.4 ± 0.3 Å), I_3_^T72V^ (20.0 ± 0.4 Å), and I_3_^F97Y^ (19.7 ± 0.2 Å). This trend of E-F distance in the I_3_ intermediate suggests that the ligand binding affinity of I_3_ increases in the order of wild type < T72V < F97Y and accounts for the trend of bimolecular rate constant for CO recombination (wild type < T72V < F97Y) determined from the kinetic analysis.

We also investigated subunit rotation angle and heme-heme distance for all the intermediates. The degree of subunit rotation is a key parameter for quantifying the degree of quaternary structural transition,^47–50,56^ and the heme-heme distance has been known to modulate cooperative ligand binding through a hydrogen-bonding network.^39^ In Fig. 5(c), the heme-heme distances in the candidate structures of I_1_, I_2_, I_3_^WT^, I_3_^T72V^, and I_3_^F97Y^ are plotted as a function of subunit rotation angle. Judging from the subunit rotation angle and the heme-heme distance, I_1_ (subunit rotation angle = −0.1 ± 0.5°, heme-heme distance = 18.0 ± 0.2 Å) and I_2_ (subunit rotation angle = 0.1 ± 0.5°, heme-heme distance = 17.9 ± 0.3 Å) have a similar structure as the initial HbI(CO)_2_ (subunit rotation angle = 0° because HbI(CO)_2_ was used as the reference structure for calculating the subunit rotation angle, heme-heme distance = 18.4 Å), indicating that the transitions from HbI(CO)_2_ to I_1_ and I_2_ accompany only minor changes in quaternary structure and hydrogen-bonding network and thus I_1_ and I_2_ have R-like structures. In contrast, all the I_3_ intermediates, I_3_^WT^ (3.5 ± 0.6°), I_3_^T72V^ (3.7 ± 1.0°), and I_3_^F97Y^ (3.0 ± 0.6°), exhibit the subunit rotation angle larger than 3°, suggesting that the formation of T-like I_3_ involves significant changes in quaternary structure and hydrogen-bonding network.

Unlike the subunit rotation angle, the heme-heme distances in the I_3_ intermediates of the three types of HbI are different from each other. I_3_^WT^ has a smaller heme-heme distance (16.6 ± 0.2 Å) than I_2_ (17.9 ± 0.3 Å) while the I_3_^T72V^ (17.8 ± 0.4 Å) and I_3_^F97Y^ (18.0 ± 0.2 Å) have similar heme-heme distances as I_2_. The similarity of the heme-heme distances in I_3_^T72V^ and I_2_ in solution is in contrast with a shorter heme-heme distance in deoxy T72V HbI (16.8 Å) than in T72V HbI(CO)_2_ (18.4 Å) observed in crystal, indicating that the T72V mutation affects the structure of T-state HbI differently depending on the phase of the environment. The larger heme-heme distance in I_3_^T72V^ and I_3_^F97Y^ than in I_3_^WT^ explains the accelerated bimolecular CO recombination in the T72V and F97Y mutants. Since I_3_^T72V^ and I_3_^F97Y^ have larger heme-heme distances than I^WT^, the entrance of CO molecules into the protein would be easier, resulting in faster CO recombination.

## V. CONCLUSION

In this work, we applied TRXSS to the T72V mutant of HbI to investigate the effect of perturbation in the interfacial water cluster on structural dynamics of the protein. From the kinetic and structural analyses of the TRXSS data, we elucidated the kinetics of protein structural transitions and the detailed structures of protein intermediates in the T72V mutant. By comparing the rates of R-T transitions for fully photolyzed and partially photolyzed forms in wild-type, F97Y, and T72V HbI, we showed that T72V HbI has higher cooperativity than wild-type and F97Y HbI. Also, by comparing the structures of protein intermediates in wild-type, F97Y, and T72V HbI, we were able to account for the difference in kinetics of protein transitions in terms of the structure of the T-like I_3_ intermediate. Especially, the shorter E-F distance and the larger heme-heme distance in I_3_^T72V^ and I_3_^F97Y^ than in I_3_^WT^ explains the accelerated CO recombination in the T72V and F97Y mutants.
